# Sequence Analysis of Two B1 Mycobacteriophages, ElvisPhasley and Mesmerelda

**DOI:** 10.17912/micropub.biology.001964

**Published:** 2026-03-22

**Authors:** Victoria Figgins, Grace Hussey, Sarah Haimowitz, Vera Pande, Marcus Royster, Emmery Flanagan, Me'Shar'li'a Fountain, Erin George, Zion Herring, Scarlett Jensen, Jenny Nguyen, Konur Onufer, Phoenix Smith, Brennan Strong, Bruce Wang, Abigail Yang, Heather Qian, Margaret Saha

**Affiliations:** 1 Department of Applied Science, William & Mary, Williamsburg, VA, US

## Abstract

Mycobacteriophages ElvisPhasley and Mesmerelda were isolated from soil and infect *Mycobacterium smegmatis* . Each phage has siphovirus morphology and encodes 102 genes. Based on broader genomic similarity to actinobacteriophages, both are assigned to the B1 subcluster.

**Figure 1. Plaque and TEM Images and Genomic Organization of ElvisPhasley and Mesmerelda:**
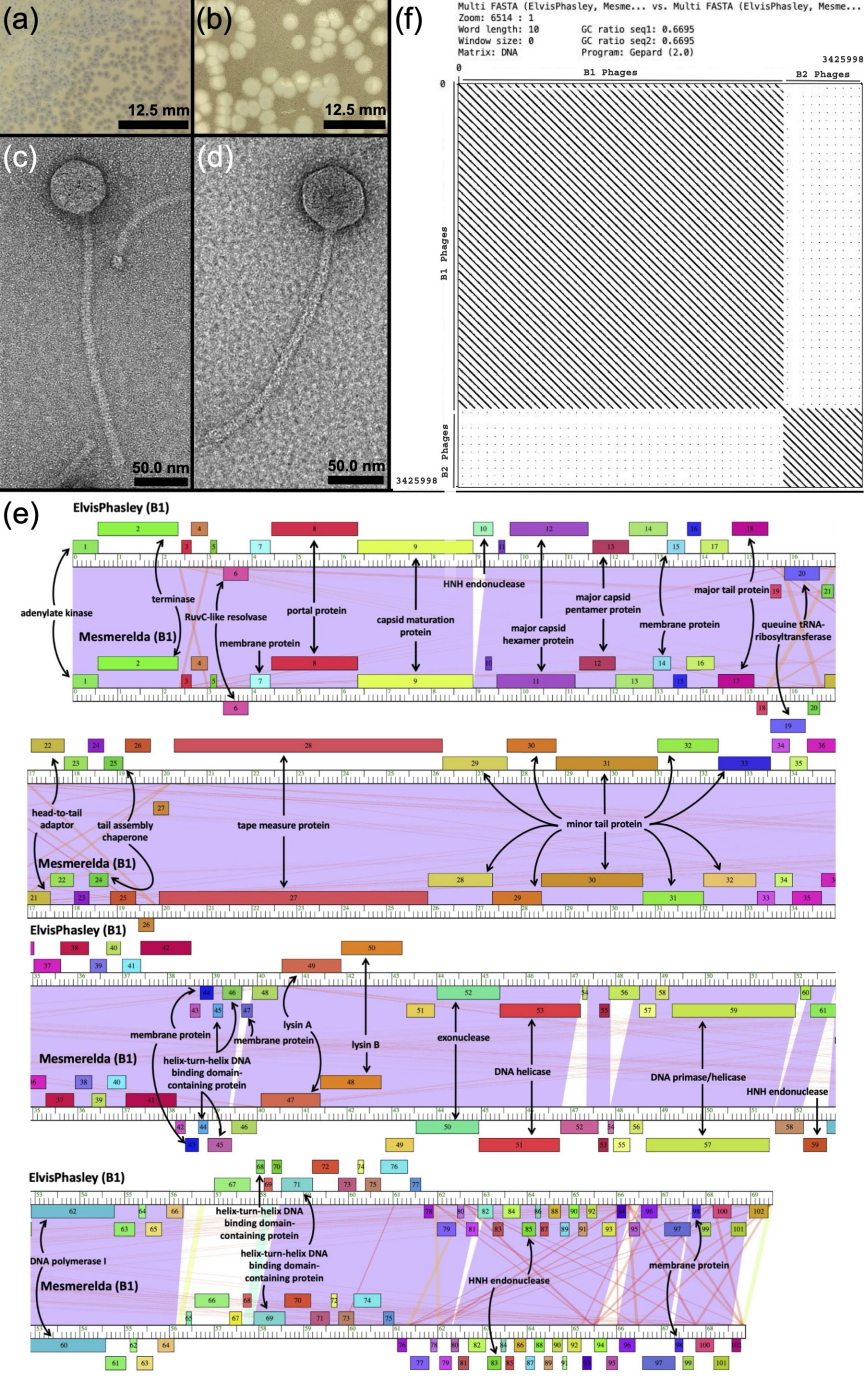
**Fig. 1** Plaque morphologies of ElvisPhasley (a) and Mesmerelda (b). ElvisPhasley forms clear plaques with translucent halos, and Mesmerelda forms clear plaques. Scale bars for plaque images are 12.5 mm. Negative stain (uranyl acetate, 1%) transmission electron microscopy (TEM) of ElvisPhasley (c) and Mesmerelda (d) revealing siphoviral morphology. Scale bars for micrographs are 50 nm. (e) Alignment of ElvisPhasley and Mesmerelda genomes using Phamerator. The genome is represented by the ruler, in kilo base pairs, with boxes above and below the ruler representing forward and reverse transcribed genes, respectively, and gene numbers presented within the box. (f) Gepard plot comparing the genomic sequences of 40 B1 cluster phages, including ElvisPhasley and Mesmerelda, and 10 B2 cluster phages. Both axes represent all 50 phage genomes, and each diagonal line on the dot plot represents high nucleotide identity between phage genome sequences. The Gepard plot exemplifies the genomic similarities of ElvisPhasley and Mesmerelda to other B1 cluster phages and the genomic dissimilarity of ElvisPhasley and Mesmerelda to B2 cluster phages.

## Description

Advancing the characterization of mycobacteriophages, bacteriophages that infect *Mycobacterium* hosts, is of interest due to increasing antibiotic resistance in pathogenic *Mycobacterium* species (Hatfull, 2020; Hatfull, 2022; Bonacorsi et al., 2024). Mycobacteriophage engineering is a promising approach in the detection and treatment of mycobacterial infections (Bonacorsi et al., 2024; Hosseiniporgham et al., 2022). Therefore, the continued characterization of mycobacteriophages broadens the capabilities of bioengineers in combating this global issue. Here, we report the genome sequences of two novel mycobacteriophages, ElvisPhasley and Mesmerelda.

Phages were isolated from two different soil samples: ElvisPhasley from wet, silty soil on the William & Mary campus in Williamsburg, VA; Mesmerelda from a bag of commercially available garden soil (Table 1). A standard enrichment procedure was followed for both samples: 5 g of each sample was suspended in 45 mL of 7H9 media, inoculated with *Mycobacterium smegmatis* mc ^2^ 155, and incubated in a 37°C shaker at 250 rpm for 48 hours (Zorawik et al., 2024). Resultant cultures were filtered using a 0.22-µm filter, and filtrates were plated in 7H9 top agar with *M. smegmatis* mc ^2^ 155. After 24-48 hours, both ElvisPhasley and Mesmerelda produced clear plaques (Fig. 1a-b). Both phages were purified with three rounds of plating, and a high titer lysate was prepared by flooding plates exhibiting nearly confluent bacterial lysis with phage buffer (10 mM Tris, pH 7.5; 10 mM MgSO _4_ ; 68 mM NaCl; 1 mM CaCl _2_ ). Negative stain (1% uranyl acetate) transmission electron microscopy of each lysate revealed siphovirus morphology for both phages (Fig. 1c-d).

Phage DNA was extracted from each high titer lysate using a phenol-chloroform-isoamyl alcohol procedure and ethanol precipitation (Sambrook and Russell, 2006). DNA was prepared for sequencing using the NEB Ultra II Library Kit and sequenced using an Illumina NextSeq 1000 sequencer (single-end, 100 base read). Raw reads were trimmed with cutadapt 4.7 (using the option: –nextseq-trim 30) and filtered with skewer 0.2.2 (using the options: -q 20 -Q 30 -n -l 50) (Martin, 2011; Jiang et al., 2014; Wick et al., 2017). Newbler (v.2.9) was then used to assemble the genome and Consed (v.29) to check for completeness (Russell, 2018; Gordon et al., 1998). Sequencing data and genome characteristics are presented in Table 1.

Genome annotation was performed using DNA Master (v.5.23.6) and PECAAN (v.20221109) (Rinehart et al., 2016; Pope and Jacobs-Sera, 2018). Start sites of protein coding genes were predicted using Glimmer (v.3.02) and GeneMark (v.2.5) (Besemer and Borodovsky, 2005; Delcher et al., 2007). These predictions were manually refined using GeneMark coding potential predictions (Besemer and Borodovsky, 2005), start site similarity comparisons between pham members obtained from Starterator ( http://phages.wustl.edu/starterator/ ), and start site alignment scores with homologous genes found using BLASTp against the NCBI non-redundant protein and Actinobacteriophage databases (Altschul et al., 1990). No tRNAs were predicted using Aragorn (v.1.2.41) and tRNAscan (Laslett and Canback, 2004; Lowe and Eddy, 1997).

Predictions from HHPred (using the PDB_mmCIF70, NCBI_CD, SCOPe70, and pFAM-A databases), BLASTp, and Phamerator for highly similar genes were used to assign putative gene functions (Cresawn et al., 2011; Zimmermann et al., 2018). Both phages are assigned to cluster B, subcluster B1 using the gene content similarity (GCS) tool at the Actinobacteriophage database, PhagesDB, and clustering parameters of at least 35% GCS to actinobacteriophages (Russell and Hatfull, 2017). Default settings were used for all software.

The genomes of ElvisPhasley and Mesmerelda both encode 102 protein-coding genes, with 33 and 32 genes, respectively, assigned functions involved in virion propagation, structure, and lysis (Fig. 1e). As neither ElvisPhasley nor Mesmerelda encode a putative integrase or other proteins implicated in lysogeny, these phages are predicted to be virulent. Both ElvisPhasley and Mesmerelda display strong genomic similarity to other B1 subcluster phages and genomic dissimilarity to B2 subcluster phages as displayed on a Gepard dot plot (Fig. 1f; Krumsiek et al., 2007). ElvisPhasley shares 89.22% GCS with its closest relative, OSMaximus (Russell and Hatfull, 2017). At the nucleotide level, ElvisPhasley shares 99.00% identity with OSMaximus over 97% coverage (BLAST). Within this covered region, we identified 968 nucleotide differences, 249 of which were found in coding regions. Of these differences, 238 resulted in amino acid substitutions, 98 of which were conservative and 140 of which were non-conservative as classified by the BLOSUM62 alignment score matrix. Likewise, Mesmerelda shares 95.1% GCS with its closest relative, Orfeu (Russell and Hatfull, 2017). These phages share 99.16% nucleotide identity over 100% coverage. Of the 557 nucleotide differences, 136 differences occur in coding regions. These differences result in 115 amino acid substitutions, 53 of which are conservative and 62 of which are not conservative.

**Nucleotide sequence accession numbers**

&nbsp;&nbsp;&nbsp;&nbsp;&nbsp;&nbsp;&nbsp;&nbsp;&nbsp;&nbsp;&nbsp; ElvisPhasley and Mesmerelda are available at GenBank with Accession No. PV876949 and PV876954 , and Sequence Read Archive (SRA) No. SRX29990110 and SRX29990101 .

**Table 1: Genome and sequencing information for Mesmerelda and ElvisPhasley**

**Table:** 

**Phage**	**ElvisPhasley**	**Mesmerelda**
Isolation GPS coordinates	37° 16' 13.4034" N 76° 43' 3.216" W	37° 16' 11.8992" N 76° 42' 52.5996" W
Morphology	Siphovirus	Siphovirus
Average capsid diameter (± SD)	60 ± 4 nm ( *n = 5* )	50 ± 4 nm ( *n = 5* )
Average tail length (± SD)	310 ± 10 nm ( *n = 5* )	220 ± 10 nm ( *n = 5* )
Sequencing reads	4,697,092	4,305,175
Sequencing coverage, fold	6,513	5,969
Genome length (bp)	69,502	68,890
Character of genome ends	Circularly permuted	Circularly permuted
Number of protein-coding genes	102	102
GC content (%)	66.4	66.4
Accession number	PV876949	PV876954
SRA	SRX29990110	SRX29990101
