# Corrigendum: Sequence Analysis of Two B1 Mycobacteriophages, ElvisPhasley and Mesmerelda

**DOI:** 10.17912/micropub.biology.002147

**Published:** 2026-04-17

## Description

**Corrigendum: Sequence Analysis of Two B1 Mycobacteriophages, ElvisPhasley and Mesmerelda**

Figgins V, et al. 2026. *microPublication Biology* . https://doi.org/10.17912/micropub.biology.001964

In the originally published article, co-author Savannah Prozik was inadvertently omitted from the author list. Her name now appears between Konur Onufer and Phoenix Smith.

